# Cheminformatics Approaches to the Analysis of Additives for Sustainable Polymeric Materials

**DOI:** 10.3390/polym17111522

**Published:** 2025-05-29

**Authors:** Alina Bărbulescu, Lucica Barbeș

**Affiliations:** 1 Department of Civil Engineering, Transilvania University of Brașov, 5 Turnului Str., 500152 Braşov, Romania; alina.barbulescu@unitbv.ro; 2 Department of Chemistry and Chemical Engineering, Ovidius University of Constanța, 124 Mamaia Bd., 900112 Constanta, Romania; 3 Doctoral School of Biotechnical Systems Engineering, Politehnica University of Bucharest, 313, Splaiul Independenţei, 060042 Bucharest, Romania

**Keywords:** sustainable polymers, additives, cheminformatics, HC, maximum common structure, similarity

## Abstract

Additives are compounds used for material to increase specific properties. When used for polymers, they extend their life and contribute to environmental sustainability. This article presents the study findings related to 24 additives—antioxidants, UV stabilizers, and quenchers—using cheminformatics methods. The compounds’ characteristics (e.g., number of atoms, functional groups) were emphasized, followed by some descriptors. The Tanimoto coefficient, computed based on the maximum common structure algorithm, and the overlap coefficient indicated the degree of similarity between the molecules. The molecules were grouped by binning and hierarchical clustering (HC) based on the extracted results. In the last case, two scenarios were considered—with four (CL1–CL4) and six clusters (CL1.1, CL1.2, CL2, CL3, CL4.1, CL4.2) being built. Considering the mechanical properties of the compounds and the standard deviation and amplitude of their values, the most homogenous class was CL2 (respectively CL4.2). Considering the toxicity of additives, the highest possible negative impact on the environment is that of the compounds in CL1 and CL3. The clustering results guide the selection of additives with reduced environmental impact, thereby supporting the development of sustainable polymer formulations aligned with circular economy principles.

## 1. Introduction

Polymeric materials are foundational to many modern applications, but their environmental impact, especially in terms of persistence and pollution, has become a pressing global issue [1]. Additives are molecules used in the fabrication of materials to improve their properties, such as durability, flexibility, or UV resistance, and their performance [2]. They can be classified, based on their function and the properties they impart to the polymeric material [3,4], into heat stabilizers (which prevent thermal degradation), UV stabilizers (which protect against the degradation caused by ultraviolet (UV) light), antioxidants, (which prevent oxidation) [5,6], flame retardants (which improve the polymers’ resistance to fire) [7], fillers (used to improve the mechanical properties, reduce cost, and enhance the specific performance characteristics of the material) [8], plasticizers (which improve the polymers’ flexibility) [9], pigments and dyes (which provide color for polymers), etc.

Significant research has been performed on various additives for polymeric materials from the viewpoint of their applications [10,11,12,13,14,15]. It has been shown that many conventional additives are derived from petrochemicals, are non-biodegradable, or exhibit toxic behavior during use or disposal [16]. Some additives, like biodegradable agents, help make plastics that are more environmentally friendly, addressing growing sustainability concerns [17]. As sustainability becomes a critical priority in materials science, there is a growing need to develop greener additives and to better understand, organize, and assess the environmental profiles of those already in use [18].

Studies indicate that different additives have potential toxicity, and have focused on clarifying the dose, concentration, and types required of various polymers to cross the organism barriers, accumulate within the organism, and ultimately damage it [1,19,20,21,22]. Hahladakis et al. [11] reviewed the main findings related to the mechanisms of release and mobility of various additives for plastics, emphasizing their effects during the lifetime of the polymeric materials in which they are incorporated.

Investigating the main exposure route to plastics, Lorca and Farré [23] showed a significant gap in the knowledge on micro- and nanoplastics toxicity to population health, resulting from a lack of in vitro studies. Such research on the molecules’ similarities was developed, especially in drug classification, to detect the candidates with specific characteristics and reduce the spectrum of experimental studies, as well as their cost and time [24,25,26]. Maggiora [27], Hu and Bajorath [28], and Stumpfe et al. [29] have pointed out compounds with high structural similarity but significant differences in their effects in terms of drug activity. Medina-Franco et al. [30] emphasized the importance of chemoinformatic software in chemical space exploration. Safizadeh et al. [31] have pointed out the use of machine learning to detect similarities in chemical structures.

The development of machine learning and statistical software such as R has provided valuable tools not only for statistical analysis but also for solving problems in engineering and decision-making [32,33,34,35,36,37,38]. Despite cheminformatics and chemometrics have proved valuable in pharmaceutical, metabonomic, and bioscience investigations [39,40,41,42], they are less utilized in other research fields.

With a large user community and a freeware license, the R software provides various packages suitable for computing molecular fingerprints and their classification [43,44]. We utilized its capabilities [45,46,47,48,49] to efficiently organize and analyze the structural characteristics of 24 additives for polymer materials. By clustering techniques, like binning and HC, the study assesses the degree of similarity [50] between the molecules, providing insights into the toxicity and chemical properties that influence polymer performance.

In transitioning toward a circular economy, using additives with low toxicity has become a strategic requirement. Classifying these additives based on molecular descriptors and toxicological properties enables the selection of those with environmentally favorable profiles, thus facilitating the design of products with extended service life and reduced environmental impact. The role of chemometric analyses and approaches based on structural descriptors in providing a solid scientific foundation for rationalizing additives cannot be overstated. Integrating these methods allows for the correlation of additive chemical compositions with relevant mechanical, thermal, and toxicological properties, directly supporting eco-design objectives and reducing ecological risks associated with polymers used in multiple life cycles [51].

The present study aligns with the Decision (EU) 2022/591 objectives on an Environment Action Programme by emphasizing additives’ essential roles in enhancing polymeric materials’ performance and facilitating their integration into a circular economic system [52]. As highlighted in recent contributions [53,54], additives such as antioxidants, UV stabilizers, compatibilizers, and pigments are not merely optional enhancements but indispensable components for maintaining the quality of recycled materials and expanding their fields of application. The integrative approach supports the competition of high-performance, low-impact additives, facilitating the design of sustainable solutions for recycling and reusing plastic materials.

## 2. Materials and Methods

The study’s stages are presented below.

1. *Install the packages necessary for the analysis and visualization from R 4.2.3.* These are chemometrics, ‘ChemmineR’, ‘cluster’, ‘factoextra’, ‘fingerprint’, ‘fmcsR’, ‘ggplot2’, ‘gridExtra’, ‘NbClust’, ‘rcdk’, ‘rgl’, and ‘vegan’.
2. *Import the compounds in R*, *in a structure definition file (SDF) format from PubChem* [55]. These are in a 2D representation, given that this is one of the most used formats in the study of similarity and neighborhood relationships [42,50]. However, we should mention that there is no best representation, as its appropriateness depends on the study’s goal [42].
3. *Create the ‘Sdfset’ instance containing all study molecules.*
4. *Visualize the compounds’ IDs and structures using the command ‘view(Sdfset)’*.

The compounds’ names (CID) are numbers assigned by the database.

5. *Plot the compounds’ structure using the command ‘plot(sdfset, …)’.*

The two-dimensional structures can be plotted in various ways emphasizing the atoms of interest or only the structure itself, without the atoms. The molecular formula (MF) and molecular weights (MWs) are also displayed. The online structure can also be visualized using the command ‘sdf.visualize(sdfset)’ from ChemMine tool [56].

6. *Detect the molecular descriptors using the ‘propma’ command from ChemmineR.*

Molecular descriptors are numbers that reflect molecules’ chemical information. According to [57], they are the results of mathematical or logical procedures that ‘transform chemical information encoded within a symbolic representation of a molecule into a useful number or the result of some standardized experiment’. These can be grouped into constitutional (e.g., atom count, bound count), topologic (e.g., longest chain, number of rings), geometric (e.g., types of rings, moments of inertia), and hybrid (e.g., shape descriptors, electro-topological index) [58]. All should be clearly defined and have invariance properties (to numbering, labeling, and roto-translation) and applicability to wide classes of molecules, structural interpretations, correlations with the experimental properties, etc. [50,59]. In another classification, the descriptors are grouped as general (based on the molecule’s representation and theoretical) or experimental (e.g., polarizability, Log P, molar refractivity) [60].

The ‘MF(sdfset)’, MW(sdfset)’, ‘atomcountMA(sdfset)’, ‘groups(sdfset)’, and ‘rings(sdfset)’ commands from ChemmineR were utilized to list the MW, MF, number and types of atoms, functional groups and rings, respectively. The library ‘ChemmineOB’ [61] was loaded and the command ‘propOB()’ was utilized to extract the values of the polar surface area (PSA), molar refractivity (MR), Log P, hydrogen bond donors (HBDs), and hydrogen bond acceptors (HBAs) [62,63].

PSA represents the surface of the polar atoms (the N and O, and H attached to them) belonging to a molecule. It is computed through the topological PSA method (TPSA) [64], which sums up the polar fragments’ surface contributions. TPSA shows a compound’s interaction potential with water and other polar media, being an indicator of the molecule’s transport capacity and toxicological properties. MR measures the polarizability of a mole of substance [65]. Log P is the logarithm of the partition coefficient and indicates the molecule’s hydrophobicity/lipophilicity [66].

7. *Evaluate the structures’ similarity using the commands from ChemmineR and ‘fmcsBatch()’ from ‘fmcsR’.*

APs consist of pairs of atoms and the lowest length of the bond path between them [67,68]. The studies [69,70] show that similarity quantification provides the number of 2D substructures common to a reference structure. A measure for similarity should incorporate (a) the representation utilized to characterize the compared molecules, (b) a scale of importance to quantify the representations’ compounds, and (c) a similarity coefficient that measures the degree of relationship between the analyzed structures [71].

Any similarity/dissimilarity measure (coefficient) is based on a specific metric (e.g., Euclidean, Manhattan, Soergel). One such measure is the Tanimoto coefficient (*TC*), whose performances were confirmed by various analyses [68,72,73], which is computed based on the molecules’ binary fingerprints, as follows per [72]:

(1) $$TC=\frac{M1\cdot M2}{\left|M1\right|+\left|M2\right|-M1\cdot M2},$$

where $\left|M1\right|$ and $\left|M2\right|$ are the numbers of bits with values 1 in the fingerprint of molecules M1, and M2, respectively, and M1·M2 is the number of bits set to 1 in both structures.

The overlap coefficient of M1 and M2 is defined by [48], as follows:

(2) $$OC=\frac{c}{min(a,b)},$$

where *a* and *b* are the number of atoms in M1 and M2, respectively, in the representation and *c* is their maximum common substructure (MCS) [38].

A version of the Tanimoto coefficient can be computed per [48], as follows:

(3) $${TC}_{M1,M2}=\frac{c}{a+b-c},$$

when considering the same principle as in the *OC* computation.

The higher the TC, the more similar the structures are. The higher the MCS, the larger the OC and ${TC}_{M1,M2}$.

8. *Cluster the Molecules*

Different methods have been used, as follows:

- Binning, which is based on the molecules’ characteristics from which a distance matrix is built. During the algorithm run, the clusters are merged using the single linkage method. Moreover, different cutoffs are provided given that the optimum cutoff is unknown [74]. This procedure is implemented in the ‘ChemmineR’ package.
- HC [75], which is implemented in the package ‘stats’ [76]. Various linkage methods have been employed (single, average, complete, ward.D2). The best one is that with the highest cophenetic correlation coefficient (Cophc) [77].

For the initial selection of the clustering method (HC or k-means) and the number of clusters, we used three internal measures—the Dunn index, connectivity, and silhouette width—and four stability measures—average distance (AD), average distance between means (ADM), average proportion of non-overlap (APN), and the figure of merit (FOM) [78], implemented in the package ‘clValid.’

In the second stage, the accuracy of the clustering obtained after running the HC (which was chosen based on the results from the first step) was assessed by bootstrapping. The average Jaccard (AvgJ) coefficient and the clusters’ instability computed after bootstrapping were used to indicate the grouping accuracy. The clustering with the highest average Jaccard (AvgJ) coefficients and the lowest instabilities was considered the best [79].

The study set consists of 24 additives, including 9 UV stabilizers, 8 antioxidants, 5 photo-stabilizers, and 2 photo-initiators, the IDs and chemical formulas of which will be presented in the next section.

## 3. Results

**Figure 1:**
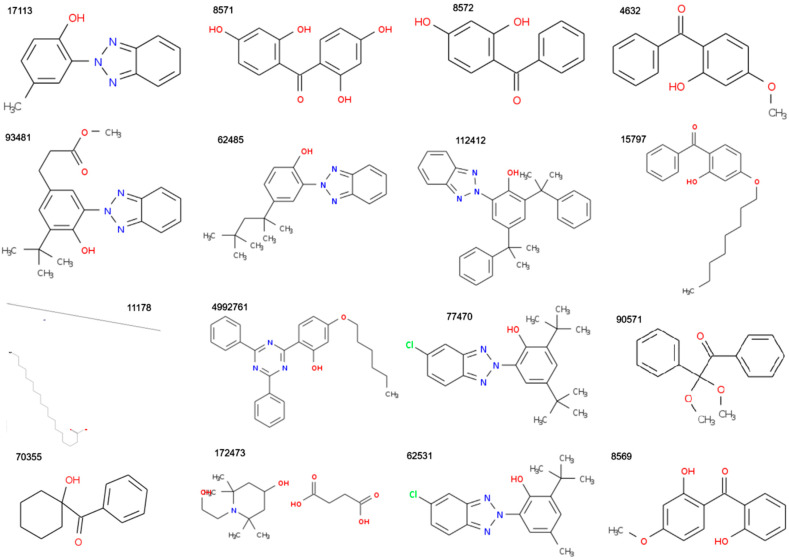
Structures of the CIDs 17113, 8571, 8572, 4632, 93481 62485, 112412, 15797, 11178, 4992761, 77470, 90571, 70355, 172473, 62531, and 8569 molecules (from top to bottom and left to right) extracted from PubChem.

**Figure 2:**
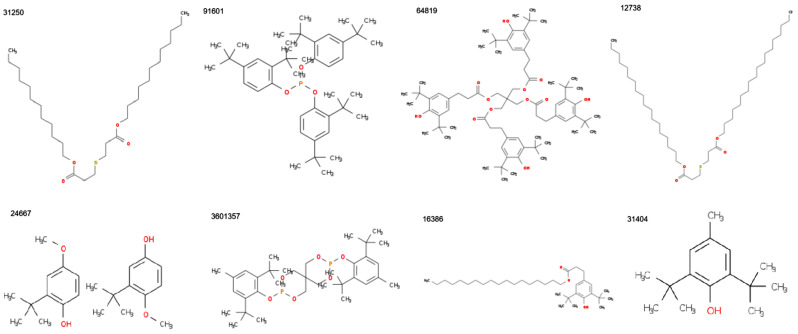
The CIDs 3125, 91601, 64819, 12738, 24667, 361357, 16386, and 31404 (from top to bottom and left to right) molecules extracted from PubChem.

The CIDs assigned in PubChem of the study molecules are presented in Figure 1 and Figure 2, together with the compounds’ structures.

**Figure 3:**
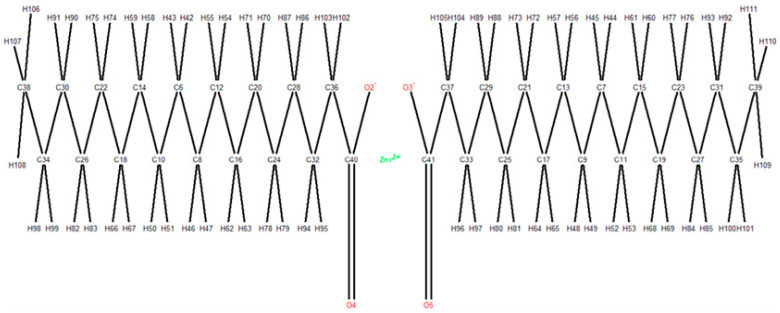
Representation of the compound with CID 11178.

Another possible representation of the molecules, indicating all atoms and their total number obtained by the internal editor of R, is shown in Figure 3 for CID 11178. This CID was chosen because the representation retrieved from PubChem is unclear. The atoms are numbered in the following order from Mendeleev’s table: from the right to the left and from the highest to the smallest atomic number.

Table 1 contains the CIDs, molecular formula (MF), molecular weight (MW), the types and number of atoms, functional groups, and rings (total and aromatics) for each compound. The number of C atoms is between 12 and 73, that of H is in the interval 10–108, and of O is between 1 and 12. Only two compounds contain P (C_42_H_63_O_3_P and C_35_H_54_O_6_P_2_), two S (C_42_H_82_O_4_S and C_42_H_82_O_4_S), one Zn (C_36_H_70_O_4_Zn), eight N (seven with three atoms and one with one atom), two S (each with one atom), and two Cl (each with one atom).

The functional groups RNH_2_, R_2_NH, ROPO_3_, RCHO, RCCH, and RCN are not found in the molecules’ structures. The functional group R_3_N appears only in C_15_H_29_NO_6_, and RCOOH is present only in C_36_H_70_O_4_Zn and C_15_H_29_NO_6_. The RCOOR group appears in CID 62819 four times, CIDs 31250 and 12738 twice, and CIDs 16383 and 93481 once. The other functional groups appear in many compounds. Rings exist in 21 components, all of which are aromatic except for CIDs 70355, 3601357, and 172473.

**Figure 4:**
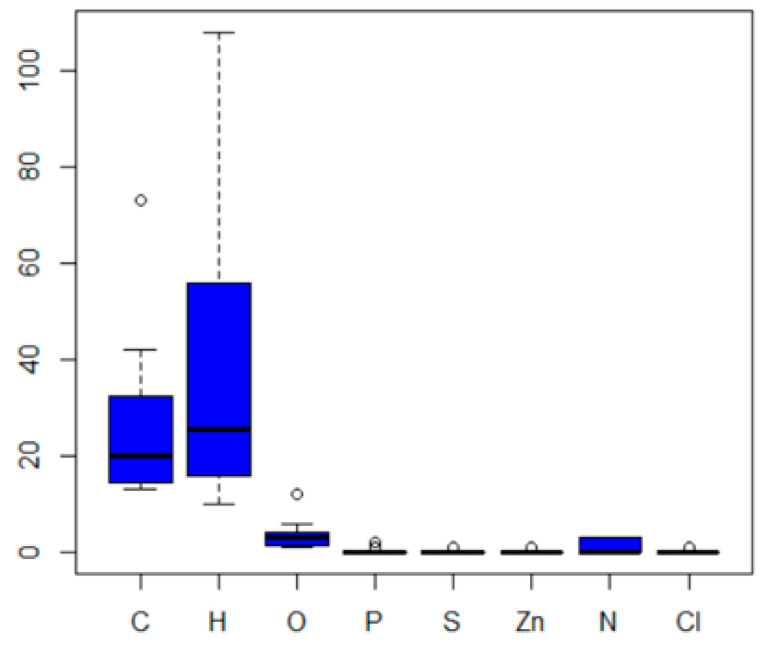
The atoms’ frequencies.

Figure 4 presents boxplots showing the atoms’ frequency. The number of H and C atoms in the set of compounds is the most variable. The distribution of P atoms in various compounds has the highest number of outliers. MR is an important physical property in polymer science that provides insights into molecular interactions, packing, and the overall behavior of the materials. It is related to the molecular volume and polarizability.

Understanding how these properties change with different molecular weights and structures can help predict how the polymer will behave in various applications. Moreover, MR can differentiate between different types of polymers and copolymers.

In the context of this study, MR could help understand how modifications in chemical structure (adding additives to the polymers) affect the overall characteristics of the material [80,81]. The highest MRs correspond to the antioxidants CID 64819 (pentaerythritol tetrakis(3-(3,5-di-tert-butyl-4-hydroxyphenyl)propionate), known as Irganox 1010); 12738 (octadecyl3-(3-octadecoxy-3-oxopropyl)sulfanylpropanoate, known as Hostanox SE 2); and 91601 (Tris(2,4-ditert-butylphenyl) phosphite, known as Irgafos 168). The lowest are associated with the UV stabilizer CID 70355 (1-Hydroxycyclohexylphenylketone) and the UV photoinitiator CID 4632 (2-hydroxy-4-methoxybenzophenone).

According to [82], systems with multiple hydrogen bonds could present high stability, given that these bonds are important in polar site binding for 2D materials. Table 2 contains molecular descriptors. HBA values up to 120 indicate a high polarity and potential for environmental mobility. The highest corresponds to CIDs 64819, 12738 (87), and 11178 (75).

HBD values in the range of 0–4 (mostly from 0 to 2, in this case) are indicative of limited reactivity and active binding. Therefore, the results from the analysis of LogP and TPSA are important to clarify their potential toxicity and environmental impact.

Lipophilicity is one of the primary indicators that help forecast the behavior and impact of chemicals in physiological and ecological systems. LogP is an important indicator in various industries targeting chemical delivery, removing substances from specific areas, and reducing environmental pollution. It should be considered in the context of the extended use of polymers for various applications, the high amount of waste containing plastics deposited in the environment, and the water pollution caused by such materials. It is directly correlated with bioaccumulation and bioconcentration factors. Evaluating LogP contributes to understanding the chemicals’ dispersion in the environment, including their bioaccumulation in soil and impact on human life [83].

In environmental risk assessment, LogP > 5 (computed for more than half of the compounds in this study) indicates that the molecules potentially accumulated in the organisms magnify up the food chain [84]. Therefore, these compounds tend to bind tightly to organic content, having low mobility but which are persistent. A LogP between 3 and 5 indicates that the molecules can pass through membranes, accumulate in tissues, and produce toxic effects. The CID 31404, 62485, 62531, 90371, and 93481 molecules have 3 < LogP < 5 and can be regarded as a ‘borderline’ concern.

The molecules CIDs 4632, 8569, 8571, 8572, 17113, and 70355, with LogP in the interval [1,3], have a mild hydrophilic–lipophilic behavior, and the compound CID 172473, with 0 < LogP < 1, has a lower lipophilicity and thus a potentially lower toxicity risk, but higher mobility. These are considered ‘safe’ based on LogP.

TPSA, though less used directly in environmental chemistry, helps estimate how easily polar compounds move through biological membranes, such as skin and fish gills. A TPSA > 140 Å^2^ [85] can give indications of reduced systemic toxicity of the molecules because they are too polar and hydrophilic to cross lipid membranes easily, penetrate cells and tissues, and be absorbed well through the gut. However, the toxicity can manifest at the contact location (e.g., skin), even if the element does not enter systemic circulation. The molecule with CID 64819 belongs in this category.

TPSA in the interval 60–140 Å^2^ shows that combining the molecules with different toxicophoric elements can result in toxic effects. With respect to the risk level, values above 75 Å^2^ indicate a higher safety when using the compounds (and thus a lower potential toxicity). The molecules with CIDs 8569, 8571, 11178, 12738, 31250, 93481, 172473, 3601357, and 4992761 belong in this category. TPSA under 60 Å^2^ indicates a higher lipophilicity and bioaccumulation property, manifested in augmented concern. Fifteen molecules belong in this category. TPSA in the interval 60–75 Å^2^ (e.g., for CIDs 8569, 11178, and 4992761) indicates a ‘borderline’ toxicity concern. These indications are not absolute. They must be analyzed in a larger context.

**Figure 5:**
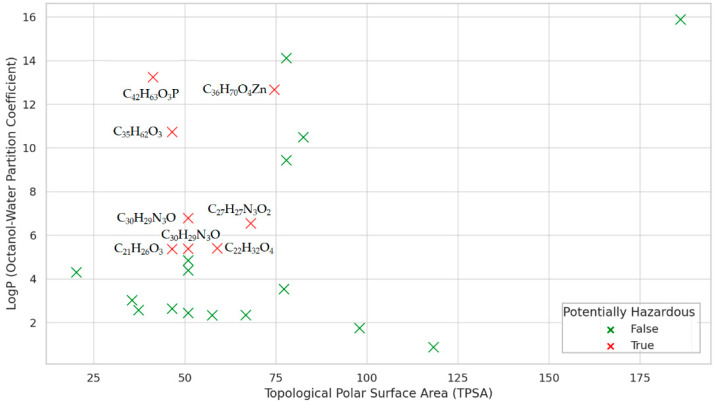
LogP vs. TPSA.

Combining the information drawn from TPSA and LogP analysis (Figure 5), we find that the molecules with LogP > 5 and TPSA < 75 Å^2^ (i.e., CIDs 11178, 15797, 16386, 24667, 77470, 91601, 112412, 4992761), flagged in red in Figure 5, are potentially hazardous because they are lipophilic and membrane-permeable, increasing the potential for bioaccumulation and systemic exposure.

Molecules 4632, 8569, 8571, 8572, 17113, 64819, 70355, and 93481 present a low environmental risk. The potential toxicity of CID 31250, 90571, and 3601357 elements (with LogP > 5 and TPSA between 77 Å^2^ and 83 Å), and CID 31404, 62485, 62531, 90571, and 93481 elements (with 3 < LogP < 5 and TPSA < 60 Å^2^), should be further investigated. For example, for CIDs 31404, 62485, and 62531, with low TPSA and moderately high LogP, possible passive uptake and moderate persistence in the environment might be possible.

Table 3 presents a triangular matrix containing the Tanimoto coefficients that evaluate the atom pairs’ similarity in different molecules. According to [24], the quantitative structure–activity relationship (QSAR) paradigm posits that molecules with high structural similarity are likely to exhibit comparable biological activities. This principle is particularly valuable when certain compounds are unavailable or prohibitively expensive, allowing for the substitution with structurally analogous alternatives.

The cutoff was set to 0.30, so only the coefficients above this level are listed. The similarity between a molecule and itself is 1, so the diagonal of the matrix contains only unitary values. Based on TC, the highest similarities are 0.76 (for the pair CIDs 4632 and 8569), 0.70 (for CIDs 4632 and 8569), and 0.66 (for CIDs 62531 and 77470), etc.

The following groups of molecules have similarities above 0.30: (4632, 8569, 8571, 8572, 15797, 17113), (11178, 12738, 31250), (17113, 62531, 77470, 93481), etc.

A deeper investigation of the molecules’ similarity was undertaken using the algorithm implemented in the fmcsR package [48], which provides the (flexible) maximum common substructure (FMCS) of pairwise compounds that contain mismatches between atoms and/or bonds. In this case, the Tanimoto coefficient was computed from graph-based MCS results, using Formula (3).

**Figure 6:**
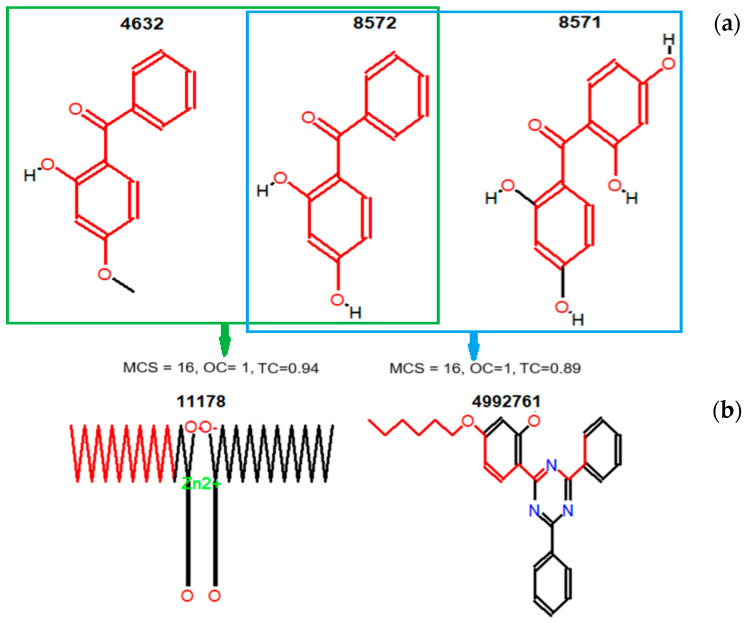
(**a**) MCS, OC, and TC for pairs (4632, 8572) and (8571, 8572) and (**b**) pair (11178, 4992761). The red part represents the MCS.

Figure 6a shows MCS = 16 for both pairs (4632, 8572) and (8571, 8572), OC = 1; the corresponding TCs are respectively 0.94 and 0.89. In Figure 6b, MCS = 7, OC = 0.22, and TC = 0.11. The TCs computed by (3) are higher than those partially presented in Table 3 (which considers only the perfect match of substructures). In the context of additive screening and design, the OC provides insights into structural compatibility and functional mimicry of candidate molecules relative to reference.

OC = 1 (Figure 6a) indicates that the entire structure of the smaller molecule is embedded within the larger one. This suggests that key functional groups or reactive centers are preserved, implying that the biological or physicochemical effects of the smaller molecule (a known additive, in this case) could be replicated or approximated by the candidate compound. A high OC (e.g., as with CIDs 62485 and 62531 or CIDs 62485 and 112412) shows significant superposition of two study structures, indicating potentially similar chemical behavior. Table 4 contains the OCs for the pairwise structures.

The first approach to clustering the compounds was binning based on their structural descriptors (Table 1 and Table 2). After normalizing the descriptors matrix, the (Euclidean) distance matrix was first built. We used a cutoff vector (0.3, 0.6). The procedure returned one cluster with two (CIDs 24667, 31404), four (CIDs 11178, 12738, 16386, and 31250), and twelve (CIDs 4632, 8569, 8571, 8572, 15797, 17113, 62485, 62531, 77470, 93481, 112412, and 4992761) elements, respectively; six clusters with one element for a cutoff of 0.3; and nineteen groups with a compound, one with two, and one with three for a cutoff of 0.6.

**Figure 7:**
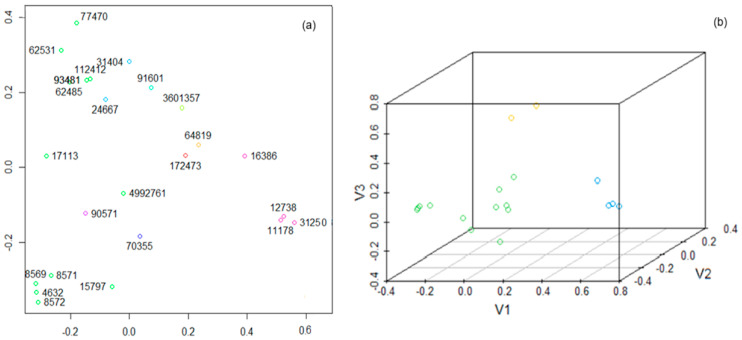
(**a**) Two-dimensional representation of the clusters obtained by binning; (**b**) three-dimensional representation of the clusters with a minimum of two elements obtained by binning. Dots of the same color represent compounds that belong to the same cluster.

Figure 7a contains the plane representation of all clusters, and Figure 7b shows the spatial representation of the clusters with a minimum of two elements when the cutoff is 0.3. Figure 7b emphasizes a good separation of the clusters with two, four, and twelve elements. The compounds 11178, 3125, 12738, and 16386 are in the same group (represented in cyclamen in Figure 7a and blue in Figure 7b). The cluster with dimension 12 (the green dots in Figure 7a) is not homogenous. Two subgroups are distinguished, i.e., (4569, 8569, 8571, 8572, 15797) and (62485, 93481, 112412, 62351), suggesting a possible refinement.

The other elements are situated at a higher distance from these subgroups, which, in turn, are not close to each other, suggesting the necessity of a deeper analysis.

To better classify the molecules, we performed clustering based on the FMCS similarity results. After running both algorithms with *k* between 2 and 4 (Table 5a) and from 2 to 6 (Table 5b), we chose the clustering algorithm (HC or k-means) based on stability and internal measures.

All measures, except Dunn, in Table 5b, indicated that the best choice is HC, so we went forward with this algorithm. To obtain the best performances in HC, the following four linkage methods were tested: ‘simple’, ‘average’, ‘complete’, and ‘Ward.D2’. The linkage method in HC was selected after observing the values of Cophc, which were 0.6924, 0.8515, 0.8637, and 0.8646, respectively. Since the highest value corresponded to the ‘Ward.D2,’ linkage, we used it in the study.

When running the algorithm with *k* between 2 and 4, we obtained the following values for the best *k*: 2 (found by AD, APM, AD, and Connectivity), 3 (provided by the Dunn index), and 4 (given by ADM, FOM, and Silhouette). Based on the majority principle, we should keep only 2 and 4 for further evaluation.

When running the algorithm with *k* between 2 and 6, ADM, APM, and FOM indicated that the best *k* = 6, while AD, Connectivity, and Silhouette indicated 5, 2, and 4, respectively. The Dunn index result was not considered as the provided result referred to *k*-means. We must analyze the case *k* = 6 based on the majority criterion.

Considering the results from Table 5, the values of interest are 2, 4, and 6. The number of clusters was decided after bootstrapping. The choice was made based on the AvgJ. A value lower than 0.65 indicates that the cluster is unstable, a value higher than 0.85 shows very high stability, and between 0.65 and 0.85 indicates stability.

For *k* = 2, AvgJ = 0.980 and 0.945 (with instabilities computed by bootstrapping of 0.026 and 0.118, respectively). For *k* = 4, the AvgJ index had values of 0.803, 0.883, 0.916, and 0.989 (with the instabilities of 0.178, 0.062, 0.035, and 0.003, respectively), so three clusters were highly stable and one stable. In the case of *k* = 6, AvgJ varied between 0.652 and 0.910, with the instabilities between 0.016 (corresponding to AvgJ = 0.910) and 0.563 (corresponding to AvgJ = 0.652). In this case, two clusters were highly stable, and the rest were stable. We also analyzed cases *k* = 3 and 5 for a comprehensive analysis, but bootstrapping revealed instability in one of the clusters in both cases.

When considering *k* = 2, the first cluster was formed by the compounds with IDs 11178, 12738, 31250, and 172473, whereas the rest were included in the second cluster. The mentioned molecules have low toxicity based on the above analysis, with the first three having a TC above 0.3 (Table 3). The last of these is situated at a certain distance in Figure 7a, indicating a higher dissimilarity with respect to the other three. This supports the idea of dividing the first cluster into two subclusters, one containing only CID 172473.

**Figure 8:**
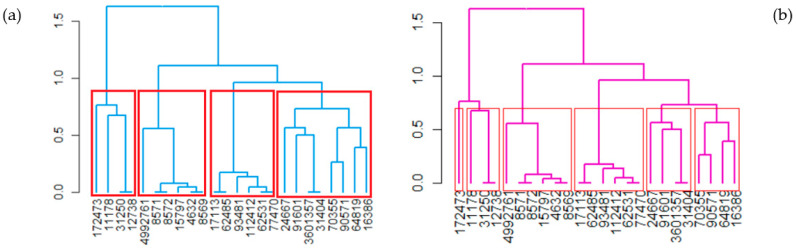
HC with Ward.D2 linkage method. (**a**) Four clusters and (**b**) six clusters.

For *k* = 4 (Figure 8a), the clusters are as follows: CL1: (11178, 12738, 31250, 172473); CL2: (4632, 8569, 8571, 8572, 15797, 4992761); CL3: (17113, 62485, 62531, 77470, 93481, 112412); and CL4: (16386, 24667, 31404, 64819, 703555, 90571, 91601, 3601357).

Grouping CIDs 4632, 8569, 8571, 8572, and 15797 in CL2 is consistent with the OC, but from the viewpoint of toxicity, the first four CIDs should belong to a cluster (no toxicity). CID is the most dissimilar with the first four molecules in this cluster, presenting the potentially highest toxicity and the lowest OC (around 0.5). Despite its potential toxicity, CID 15797 has an OC = 1 with respect to CID 4632, supporting its presence in CL2.

CL3 is the most homogenous, with OC values above 0.94 and high MR (all but CID 17113, which also has a potentially lower toxicity risk). CL4 contains molecules with various potential toxicity levels, some of which are flagged—CIDs (16386 and 64819), and (24667, 91601)—others with a low level (3601357 and 70355), and others deserving a deeper investigation.

When *k* = 6, the second and third clusters remain the same as when *k* = 4, but CL1 and CL4 are split into two clusters each. As a result, we have Cl1.1 = (172473) and CL1.2 = (11178, 12738, 31250) instead of CL1, and CL4.1 = (24667, 31404, 91601, 3601357) and CL4.2 = (16386, 64819, 70355, 90571) instead of CL4 (Figure 8b). The grouping of the molecules in CL4.1 is consistent with that shown in Figure 7a, where the distances between them are relatively low. In contrast, the molecules in CL4.2 are situated at higher distances in separate clusters obtained by binning.

**Figure 9:**
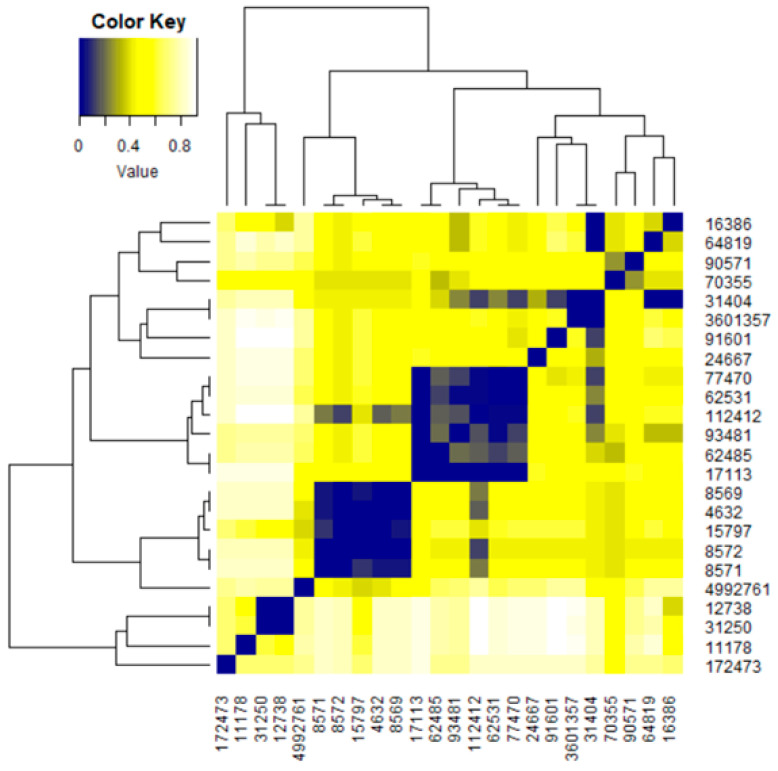
Heatmap. The darker the color, the lower the dissimilarity.

The heatmap (Figure 9) indicates the dissimilarities between the compounds. The darker the color, the lower the dissimilarity. On the diagonals, squares in dark blue indicate that the corresponding compounds are very similar, e.g., 4632, 8569, 70355, 8571, 8572, and 15797. With a higher degree of dissimilarity compared with the previous group, we find CID 4992761 in its neighborhood.

Figure 9 shows that CL3 is the most homogenous cluster (having the smallest height of the corresponding sub-tree), followed by CL2. The most inhomogeneous is CL1, which is composed as follows:

- A quencher—butanedioic acid;1-(2-hydroxyethyl)-2,2,6,6-tetramethylpiperidin-4-ol (HALS GW 622—CID 172473)—CL1.1;
- A heat stabilizer—zinc stearate (CID 11178)—CL1.2;
- Two antioxidants—dodecyl 3-(3-dodecoxy-3-oxopropyl)sulfanylpropanoate (Irganox PS 800—CID 31250) and octadecyl 3-(3-octadecoxy-3-oxopropyl)sulfanylpropanoate (Songnox^®^DSTDP—CID 12738)—CL1.2.

CL2 contains five UV stabilizers, which are 2-hydroxy-4-methoxybenzophenone (CID 4632); 2,2′-dihydroxy-4-methoxybenzophenone (CID 8569); 2,4-dihydroxybenzophenone (CID 8572); 2-hydroxy-4-n-octoxybenzophenone (CID 15797); and o-toluidine (4992761). The same group contains a UV absorber—bis(2,4-dihydroxyphenyl)methanone (MAXGARD 1000—CID8571).

CL3 is formed as follows:

- Four UV stabilizers, as follows: CID 17113—2-(2′-hydroxy-5′-methylphenyl)-benzophenone; CID 62531—(2′-Hydroxy-3′-tert-5′-methylphenyl)-5-chlorobenzotriazole; CID 77470—2-(2′-hydroxy-3′,5′-di-tert-butylphenyl)-5-chlorobenzotriazole; and CID 112412—2-(2H-Benzotriazol-2-yl)-4,6-bis (1-methyl-1-phenylethyl) phenol;
- Two UV absorbers: CID 62485—2-(benzotriazol-2-yl)-4-(2,4,4-trimethylpentan-2-yl)phenol (SUNSORB 5411)—and CID 93481—methyl 3-[3-(benzotriazol-2-yl)-5-tert-butyl-4-hydroxyphenyl]propanoate (TINUVIN 1130).

CL4.1 contains only antioxidants: Butylated hydroxyanisole (BHA—CID 24667), 2,6-Di-tert-butyl-4-methylphenol (BHT—CID 31404), Tris(2,4-ditert-butylphenyl) phosphite (ALKANOX 240—CID 91601), and Bis (2,6-di-ter-butyl-4-methylphenyl) pentaerythritol-diphosphite (ADK STAB PEP-36—CID 3601356).

CL4.2 is formed by two antioxidants—Irganox 1010 and 1076 (CIDs 64819 and 16386) and two photonitiators (1-hydroxycyclohexylphenylketone—CID 70355—and 2,2-Dimethoxy-2-phenylacetophenone—CID 90571).

## 4. Discussion

The evaluation of the toxicity of additives based on their chemical structure through the analysis of molecular descriptors (HBA, HBD, LogP, MR, TPSA) provides insights into how these additives may interact with the biological environment. For example, molecules with a high number of hydrogen bond acceptors and donors (HBAs and HBDs) may have increased bioavailability. Therefore, they can interact more easily with biological receptors, increasing their toxic potential. In this regard, compounds with high HBA, such as CID 64819 (HBA = 120) and CID 12738 (HBA = 87), could pose a greater toxic risk due to their ability to form multiple interactions with biomolecules.

LogP is a critical parameter for assessing the hydrophobicity of molecules and their potential to bioaccumulate in the tissues of living organisms. Elevated LogP values—such as those observed for CID 64819 (LogP = 15.8792) and CID 12738 (LogP = 14.1092)—indicate a strong tendency for accumulation in lipid-rich tissues, which may contribute to long-term toxic effects. This characteristic is particularly relevant in the context of polymer and plastic additives, as these substances can leach from discarded plastic products, enter the environment, and subsequently infiltrate the food chain.

Beyond hydrophobicity, the toxicological profile of additives can also be inferred from the presence of specific functional groups. Functional groups, such as tertiary amines (R_3_N), hydroxyl groups (ROH), and carboxylic acids (RCOOH), are known to engage in biologically significant interactions. For example, compounds containing tertiary amine groups—such as CID 172473 (C_15_H_29_NO_6_)—may interact with enzymatic systems and are associated with potential neurotoxic effects. Similarly, the presence of carboxylic acid groups, as identified in CID 11178 and CID 172473, may indicate a risk of irritating or corrosive effects upon exposure. Furthermore, phosphorus-containing compounds such as CID 3601357 and CID 91601, commonly employed as antioxidants and stabilizers, may raise concerns regarding hepatotoxicity and nephrotoxicity, underscoring the importance of comprehensive toxicological evaluation.

The analysis of Table 1 indicates that certain molecules contain Zn, S, and Cl atoms (C_17_H_18_ClN_3_O) that may contribute to increased human toxicity or environmental impact. CID 11178 (C_36_H_70_O_4_Zn) contains zinc, an element that can have negative effects on aquatic organisms. CID 12738 (C_42_H_82_O_4_S) and CID 31250 (C_30_H_58_O_4_S) contain sulfur, which may make them potential pollutants for aquatic environments, especially in the presence of other heavy metals. These compounds belong to CL1.2. CID 62531 (C_17_H_18_ClN_3_O) and CID 77470 (C_20_H_24_ClN_3_O) from CL3 contain chlorine, making them susceptible to generating toxic metabolites under oxidative conditions.

Additives with high MR values, such as CID 64819 (MR = 348.9130) and CID 12738 (MR = 214.1690), possess significant electronic polarizability, suggesting a pronounced ability to interact with the polymer chains at the molecular level. While these interactions may enhance mechanical properties and stability, they also raise concerns about biocompatibility and environmental risks. Additives with high MR may become more firmly embedded within the polymer matrix, complicating their degradation or removal. Over time, microparticulate release or leaching—especially under heat, UV radiation, or mechanical stress—can introduce these substances into the surrounding environment or biological systems. Moreover, the polarizability associated with high MR compounds may also influence molecular recognition and binding affinities in biological contexts, potentially exacerbating their interaction with cellular membranes. This can heighten the risk of bioaccumulation, particularly if the compound exhibits other hazardous structural features, such as reactive functional groups or high lipophilicity (e.g., high LogP).

The quality of polymeric materials containing additives and undergoing oxidative degradation under different natural environmental conditions (e.g., solar radiation, saline environments, soil exposure) was assessed by testing a series of phenolic and phosphorus compounds, such as Irganox (also studied here) [14,86]. The quantitative characterization of the effective use of additives and the evaluation of polypropylene (PP) stability under oxidation were successfully performed using ATR-FTIR spectrometry. Based on the spectrometric results, carbonyl indices (indicators of polymer material oxidation) were calculated for each type of polymer matrix containing varying amounts of additives (e.g., Irganox 1010, calcium stearate, etc., in concentrations ranging from 1% *w*/*w* to 3% *w*/*w*) over different time intervals. Low-density polyethylene (LDPE)-based polymeric composites containing additives were subjected to mechanical testing to evaluate their behavior following exposure to atmospheric conditions over 120 days. Key mechanical properties—such as tensile strength, elasticity modulus, impact resistance, and melt flow index (MFI)—were analyzed, highlighting the influence of additive concentration on the durability and long-term stability of the materials. The results confirm the hypothesis that additives improve specific mechanical properties and delay the degradation processes caused by UV radiation and other environmental factors [87], but it is also important to consider the environmental effects.

Reformulation with functional additives has become an essential practice to maintain the functionality and processability of polymeric composites. It has been shown that adding a synergistic antioxidant system (e.g., Irganox 1010 and Irgafos 168) can effectively prevent thermal oxidation during the processing of polymeric waste [88]. Compatibilization based on modified polyolefins (e.g., polypropylene grafted with maleic anhydride) is crucial for preserving good interfacial adhesion in composite materials derived from mixed waste streams [89].

A comparative analysis of the basic statistics of the mechanical properties of the elements in clusters CL1–CL4 is presented in Table 6. CL1 (CL3) elements have the smallest (highest) minimum and average tensile strength, bending elasticity, impact resistance, and melting flow. The highest (smallest) maxima and standard deviations correspond to CL4 (CL2). As a result, the highest variations of the compounds’ mechanical properties are in CL4 and the lowest in CL2. This finding is confirmed by the values of the amplitude (difference between maximum and minimum) of the characteristics, as follows: 4 MPa (tensile strength), 120 MPa (bending elasticity modulus), 0.60 kJ/m^2^ (impact resistance), and 0.40 g/10 min (melting flow index) in CL2. These are compared with the following from CL4: 8 MPa (tensile strength), 315 MPa (bending elasticity modulus), 1.65 kJ/m^2^ (impact resistance), and 0.90 g/10 min (melting flow index). Moreover, this remark supports the idea of the high inhomogeneity of CL4 and its split in CL4.1 and CL4.2.

Table 7 presents the basic statistics of the mechanical characteristics in the clusters obtained after splitting CL1 and CL4 according to HC with *k* = 6. In this case, CL4.2 is the most homogenous among these subclusters, with minimum standard deviations and amplitudes of bending elasticity modulus (215 MPa), impact resistance (1.5 kJ/m^2^), and melting flow index (0.60 g/10 min).

The values corresponding to the elements in CL1.1 are closer to the minimum in CL1.2, justifying the idea that its extraction from CL1 increased the standard deviations and averages. Despite CL4.1 being formed only by antioxidants, it presents a relatively high dissipation of the values of mechanical characteristics.

Research has shown that phosphorus-based antioxidant additives (e.g., tris(nonylphenyl) phosphate) are employed in polyolefin systems to inhibit thermal oxidation and extend the service life of polymers. They play a critical role in stabilizing polymers against degradation during both initial processing and subsequent thermal exposure. However, under repeated mechanical recycling conditions, these compounds are subject to partial depletion and chemical transformation. Empirical studies have shown that after approximately three to five recycling cycles, the concentration of phosphorus-based antioxidants can diminish by 40–60% due to thermal and oxidative degradation processes [90], leading to the formation of secondary by-products that may adversely interact with other stabilizing agents, particularly calcium and zinc stearates, which are commonly used as thermal stabilizers. Notably, phosphorus compounds have been reported to form complex metal–phosphorus species during reprocessing, which can disrupt the intended distribution and function of calcium/zinc stabilizers [91]. As a consequence, a measurable decline in thermal stability is observed, thermogravimetric analysis (TGA) indicating a 15–25% reduction in resistance to thermal degradation following multiple recycling cycles. Beyond thermal performance, these additive interactions and degradation processes significantly impact the mechanical integrity of the recycled material. Chemical alterations at the molecular level contribute to increased polymer chain scission, leading to enhanced brittleness and a reduction in tensile and impact strength. Quantitative assessments have demonstrated a mechanical performance loss of up to 30% after three recycling cycles [92]. Furthermore, these degradation pathways may induce processing challenges during re-extrusion or molding, such as inconsistent melting behavior and elevated emission of volatile or toxic by-products, raising environmental and occupational health concerns and compromising the quality and safety of the final recycled product [93].

To emphasize the practical relevance of cheminformatics in the context of the circular economy (CE), Table 8 presents a synthesis of the correspondences between the methodological components used in this study (molecular descriptor extraction, structural similarity analysis, additive classification) and the key objectives of the CE as applied to polymeric materials.

A holistic approach is required that supports the advanced sustainability of materials, efficient and continuous resource improvement, and waste reduction in polymer recycling processes to highlight the importance of the correlation between the chemometric analysis components of polymer additives and the CE objectives. By integrating chemometric techniques, our study is closely aligned with the principles of the CE and promotes both ecological and economic benefits.

## 5. Conclusions

The present article presents the application of chemometric methods in analyzing some additives for polymers. The study reveals a high HBA count (from 13 to 120) and low HBD values (mostly 0–2). This pattern reflects the structural design of these molecules, which favor high polarity and low reactivity to enhance compatibility and stability in polymer matrices. While high HBA and low HBD values suggest poor membrane permeability and reduced potential for systemic bioavailability, they do not preclude toxicological concern. Some additives exhibit high LogP and molar refractivity (MR), which may offset polar characteristics and favor bioaccumulation or chronic toxicity. Therefore, these descriptors indicate that additives may have low acute toxicity via passive diffusion but could still pose environmental or receptor-mediated risks, warranting further analysis within QSAR or regulatory frameworks.

While binning clustering grouped the molecules based on their descriptors and the 2D and 3D representations gave an image of the molecules’ similarities, HC (chosen based on internal and stability measures, and validated by bootstrapping) provided a more appropriate classification. Based on this, the compounds were classified into either four or six groups. The highest homogeneity from the viewpoint of the mechanical characteristics, evaluated using the standard deviation and amplitude, was that of the elements in the second cluster (when *k* = 4) of cluster C4.2 (when *k* = 6). The toxicity analysis indicates that the compounds with the highest toxicity were those from the first and third clusters.

The information obtained from the above analysis is helpful in the selection and combination of additives in polymer formulations, optimizing their performance according to the desired applications (UV protection, thermal stabilization, durability, etc.). The analysis could be extended by including other categories of additives (hardeners, compatibilizers, antifungals, antibacterials, or biocides) to obtain a complete picture of the behavior of these chemicals in the polymer matrix.

In the future, we intend to use more descriptors and molecular simulation methods (e.g., docking, DFT, molecular dynamics) to analyze the interactions between additives and polymer chains and evaluate their impact on the mechanical and thermal properties of materials. Moreover, the experimental research which is currently in progress will complete the knowledge provided by the above results.

## Figures and Tables

**Table 1:** The compounds’ characteristics.

MF	CID	MW	C	H	O	P	S	Zn	N	Cl	R3N	ROH	RCOR	RCOOH	RCOOR	ROR	Rings	Aromatics
C_14_H_12_O_3_	4632	228.24	14	12	3	0	0	0	0	0	0	1	1	0	0	1	2	2
C_14_H_12_O_4_	8569	244.24	14	12	4	0	0	0	0	0	0	2	1	0	0	1	2	2
C_13_H_10_O_5_	8571	246.22	13	10	5	0	0	0	0	0	0	4	1	0	0	0	2	2
C_13_H_10_O_3_	8572	214.21	13	10	3	0	0	0	0	0	0	2	1	0	0	0	2	2
C_36_H_70_O_4_Zn	11178	632.35	36	70	4	0	0	1	0	0	0	0	0	2	0	0	0	0
C_42_H_82_O_4_S	12738	683.16	42	82	4	0	1	0	0	0	0	0	0	0	2	0	0	0
C_21_H_26_O_3_	15797	326.42	21	26	3	0	0	0	0	0	0	1	1	0	0	1	2	2
C_35_H_62_O_3_	16386	530.86	35	62	3	0	0	0	0	0	0	1	0	0	1	0	1	1
C_13_H_11_N_3_O	17113	225.24	13	11	1	0	0	0	3	0	0	1	0	0	0	0	3	3
C_22_H_32_O_4_	24667	360.48	22	32	4	0	0	0	0	0	0	2	0	0	0	2	2	2
C_30_H_58_O_4_S	31250	514.84	30	58	4	0	1	0	0	0	0	0	0	0	2	0	0	0
C_15_H_24_O	31404	220.35	15	24	1	0	0	0	0	0	0	1	0	0	0	0	1	1
C_20_H_25_N_3_O	62485	323.43	20	25	1	0	0	0	3	0	0	1	0	0	0	0	3	3
C_17_H_18_ClN_3_O	62531	315.79	17	18	1	0	0	0	3	1	0	1	0	0	0	0	3	3
C_73_H_108_O_12_	64819	1177.63	73	108	12	0	0	0	0	0	0	4	0	0	4	0	4	4
C_13_H_16_O_2_	70355	204.26	13	16	2	0	0	0	0	0	0	1	1	0	0	0	2	1
C_20_H2_4_ClN_3_O	77470	357.88	20	24	1	0	0	0	3	1	0	1	0	0	0	0	3	3
C_16_H_16_O_3_	90571	256.29	16	16	3	0	0	0	0	0	0	0	1	0	0	2	2	2
C_42_H_63_O_3_P	91601	646.92	42	63	3	1	0	0	0	0	0	0	0	0	0	0	3	3
C_20_H_23_N_3_O_3_	93481	353.41	20	23	3	0	0	0	3	0	0	1	0	0	1	0	3	3
C_30_H_29_N_3_O	112412	447.57	30	29	1	0	0	0	3	0	0	1	0	0	0	0	5	5
C_15_H_29_NO_6_	172473	319.39	15	29	6	0	0	0	1	0	1	2	0	2	0	0	1	0
C_35_H_54_O_6_P_2_	3601357	632.75	35	54	6	2	0	0	0	0	0	0	0	0	0	0	4	2
C_27_H_27_N_3_O_2_	4992761	425.52	27	27	2	0	0	0	3	0	0	1	0	0	0	1	4	4

**Table 2:** The molecular descriptors.

CID	HBA	HBD	LogP	MR	TPSA	CID	HBA	HBD	LogP	MR	TPSA
4632	15	1	2.6318	64.8315	46.53	62485	28	1	4.8399	99.1260	50.94
8569	16	2	2.3374	66.8545	66.76	62531	21	1	4.3854	90.1340	50.94
8571	15	4	1.7400	64.4085	57.99	64819	120	4	15.8792	348.9130	186.12
8572	13	2	2.3288	60.3625	57.53	70355	18	1	2.5645	59.7573	37.30
11178	75	2	12.6625	180.8236	74.60	77470	27	1	5.3745	104.4380	50.94
12738	87	0	14.1092	214.1690	77.90	90571	19	0	3.0151	72.7905	35.53
15797	29	1	5.3625	98.4805	46.53	91601	67	0	13.2353	203.7500	41.28
16386	65	1	10.7245	169.3960	46.53	93481	28	1	3.5292	100.8300	77.24
17113	14	1	2.4345	65.8540	50.94	112412	32	1	6.7779	138.7880	50.94
24667	36	2	5.3966	108.4540	58.92	172473	36	4	0.8663	86.9732	118.30
31250	63	0	9.4280	156.4850	77.90	3601357	62	0	10.4850	180.9930	82.56
31404	25	1	4.2956	71.9710	20.23	4992761	32	1	6.5373	128.6850	68.13

**Table 3:** TCs higher than 0.3.

CID	4632	8569	8571	8572	11178	12738	15797	16386	17113	24667	31250	31404	62485	62531	77470	93481	112412	4992761
4632	1.00	0.76	0.42	0.70			0.42		0.33									
8569		1.00	0.55	0.60			0.35		0.33									
8571			1.00	0.57														
8572				1.00			0.36		0.33									
11178					1.00	0.30					0.42							
12738						1.00					0.52							
15797							1.00											0.34
16386								1.00			0.30							
17113									1.00				0.44	0.44	0.30	0.32		
24667										1.00		0.45						
31250											1.00							
31404												1.00						
62485													1.00	0.43	0.42	0.41		
62531														1.00	0.66	0.47	0.30	
77470															1.00	0.44	0.37	
93481																1.00		
112412																	1.00	
4992761																		1.00

**Table 4:** The OCs computed using the fmcsR package.

CID	4632	8569	8571	8572	11178	12738	15797	16386	17113	24667	31250	31404	62485	62531	64819	70355	77470	90571	91601	93481	112412	172473	3601357	4992761
4632	1	1	0.94	1	0.18	0.18	1	0.53	0.47	0.53	0.18	0.56	0.53	0.53	0.59	0.6	0.53	0.53	0.53	0.53	0.82	0.18	0.53	0.59
8569		1	0.94	1	0.17	0.17	0.94	0.5	0.47	0.5	0.17	0.56	0.5	0.5	0.61	0.6	0.5	0.5	0.5	0.5	0.78	0.17	0.5	0.56
8571			1	1	0.17	0.17	0.89	0.5	0.47	0.5	0.17	0.56	0.5	0.5	0.56	0.6	0.5	0.5	0.5	0.5	0.78	0.17	0.5	0.5
8572				1	0.19	0.19	1	0.56	0.5	0.56	0.19	0.56	0.56	0.56	0.63	0.6	0.56	0.56	0.56	0.56	0.88	0.19	0.56	0.56
11178					1	0.46	0.38	0.5	0.12	0.12	0.37	0.19	0.21	0.14	0.17	0.47	0.12	0.21	0.1	0.23	0.09	0.32	0.12	0.22
12738						1	0.42	0.61	0.12	0.12	1	0.19	0.21	0.14	0.19	0.47	0.12	0.26	0.09	0.23	0.09	0.32	0.12	0.25
15797							1	0.42	0.47	0.38	0.42	0.56	0.38	0.41	0.42	0.6	0.38	0.47	0.38	0.38	0.58	0.32	0.38	0.63
16386								1	0.47	0.42	0.49	1	0.42	0.55	0.61	0.6	0.56	0.47	0.37	0.65	0.41	0.32	0.42	0.25
17113									1	0.41	0.12	0.5	1	1	0.47	0.47	1	0.41	0.47	1	1	0.12	0.47	0.47
24667										1	0.12	0.69	0.42	0.5	0.42	0.53	0.44	0.42	0.42	0.42	0.42	0.18	0.42	0.31
31250											1	0.19	0.21	0.14	0.26	0.47	0.12	0.26	0.11	0.23	0.09	0.32	0.14	0.25
31404												1	0.63	0.75	1	0.53	0.88	0.5	0.88	0.75	0.88	0.25	1	0.5
62485													1	0.86	0.42	0.67	0.83	0.47	0.46	0.79	0.83	0.27	0.42	0.33
62531														1	0.55	0.53	1	0.42	0.55	0.95	0.95	0.18	0.55	0.36
64819															1	0.6	0.56	0.47	0.3	0.65	0.41	0.27	0.37	0.25
70355																1	0.53	0.73	0.6	0.6	0.53	0.47	0.6	0.47
77470																	1	0.42	0.6	0.88	0.96	0.18	0.56	0.32
90571																		1	0.42	0.47	0.42	0.26	0.42	0.37
91601																				0.5	0.44	0.18	0.4	0.25
93481																				1	0.85	0.27	0.46	0.31
112412																					1	0.18	0.41	0.25
172473																						1	0.27	0.27
3601357																							1	0.25
4992761																								1

**Table 5:** Stability and internal measures for clustering.

Stability			Internal		
(a) Run from two to four clusters					
Measures	Score	No of clusters	Measures	Score	No of clusters
AD	0.7709	2	Connectivity	4.8409	2
ADM	0.0455	4	Dunn	0.6880	3
APM	0.0231	2	Silhouette	0.3969	4
FOM	0.1661	4			
(b) Run from two to six clusters					
Measures	Score	No of clusters	Measures	Score	No of clusters
AD	0.5655	5	Connectivity	4.8409	2
ADM	0.0232	6	Dunn	0.7910	6 (k-means)
APM	0.0089	6	Silhouette	0.3969	4
FOM	0.1175	6			

**Table 6:** Basic statistics of the mechanical properties of the compounds in CL1–CL4.

Cluster	Statistics	Tensile Strength (MPa)	Bending Elasticity Modulus (MPa)	Impact Resistance (kJ/m^2^)	Melting Flow Index (g/10 min)
CL1	Min	19.00	1100.00	3.20	2.30
	Max	26.00	1375.00	4.30	3.10
	Mean	22.00	1218.75	3.68	2.65
	Std.dev.	2.55	105.14	0.42	0.30
CL2	Min	21.00	1180.00	3.50	2.60
	Max	25.00	1300.00	4.10	3.00
	Mean	23.00	1235.00	3.78	2.80
	Std.dev.	1.41	43.30	0.22	0.14
CL3	Min	22.00	1230.00	3.60	2.70
	Max	29.00	1450.00	4.60	3.50
	Mean	25.67	1341.67	4.13	3.10
	Std.dev.	2.50	83.29	0.37	0.29
CL4	Min	22.00	1185.00	3.50	2.60
	Max	30.00	1500.00	5.00	3.50
	Mean	25.50	1332.50	4.18	2.98
	Std.dev.	2.95	110.44	0.52	0.32

**Table 7:** Basic statistics of the mechanical properties of the compounds in CL1.1, CL1.2, CL4.1, and CL4.2.

Cluster	Statistics	Tensile Strength (MPa)	Bending Elasticity Modulus (MPa)	Impact Resistance (kJ/m^2^)	Melting Flow Index (g/10 min)
CL1.1	Value	21.00	1150.00	3.40	2.50
CL1.2	Min	19.00	1100.00	3.20	2.30
	Max	26.00	1375.00	4.30	3.10
	Mean	22.33	1241.67	3.77	2.70
	Std.dev.	2.87	112.42	0.45	0.33
CL4.1	Min	25.00	1150.00	3.40	2.50
	Max	30.00	1500.00	5.00	3.50
	Mean	27.50	1400.00	4.50	3.15
	Std.dev.	3.54	141.42	0.71	0.49
CL4.2	Min	22.00	1185.00	3.50	2.60
	Max	28.00	1400.00	4.50	3.20
	Mean	24.50	1298.75	4.03	2.90
	Std.dev.	2.52	95.43	0.43	0.24

**Table 8:** Correlation of chemometric analysis and the objectives of the CE.

Component	Description	Objective	References
Additive classification based on structural or functional similarity	Molecular descriptor-based clustering (e.g., FMCS, Tanimoto coefficient)	Enhancement of recycling formulations via the identification of additives with analogous behavior in polymers	[27,41,53]
Computation of descriptors (LogP, MR, HBA, HBD, TPSA)	Assessment of toxicological potential, molecular polarizability, and bioaccumulation capacity	Minimizing ecological and toxicological risks when choosing additives	[64,65,85]
Detection of high-performance mechanical clusters	Structure–property correlation with respect to key physico-mechanical parameters (e.g., MFI, elasticity)	Enhancement of the durability and lifespan of products and materials	[80,81,85]
Screening for additives with structural compatibility	Classification of additives according to their functional applicability (e.g., UV stabilizers, antioxidants)	Smart reuse strategies and function-driven design	[3,10,13]
Removal of potentially dangerous compounds (e.g., derivatives based on Cl, Zn, S)	Chemical structure and ecotoxicity-related molecular descriptors	Hazardous waste elimination and the promotion of alternative substitution	[11,12,94]

## Data Availability

Data are available for downloading at https://pubchem.ncbi.nlm.nih.gov/ (accessed on 10 March 2025).

## Abbreviations

The following abbreviations are used in this manuscript:

AD	Average distance
ADM	Average distance between means
APN	Average proportion of non-overlap
AvgJ	Average Jaccard coefficient
CID	Compound identification name
CE	Circular economy
Cophc	Cophenetic correlation coefficient
FOM	Figure of merit
HBA	Hydrogen bond acceptor
HBD	Hydrogen bond donors
Log P	Logarithm of the partition coefficient
MF	Molecular formula
MR	Molar refractivity
MW	Molecular weight
PSA	Polar surface area
SAR	Structure-activity relationship
SDF	Structure definition file
TPSA	Topological PSA
